# Evaluation of a Low-Dose Computed Tomography Lung Cancer Screening Program in Henan, China

**DOI:** 10.1001/jamanetworkopen.2020.19039

**Published:** 2020-11-03

**Authors:** Lan-Wei Guo, Qiong Chen, Yin-Chen Shen, Qing-Cheng Meng, Li-Yang Zheng, Yue Wu, Xiao-Qin Cao, Hui-Fang Xu, Shu-Zheng Liu, Xi-Bin Sun, You-Lin Qiao, Shao-Kai Zhang

**Affiliations:** 1 Henan Engineering Research Center of Cancer Prevention and Control, Henan International Joint Laboratory of Cancer Prevention, Department of Cancer Epidemiology and Prevention, Henan Cancer Hospital, The Affiliated Cancer Hospital of Zhengzhou University, Zhengzhou, China; 2 Office of Cancer Screening, National Cancer Center, National Clinical Research Center for Cancer, Cancer Hospital, Chinese Academy of Medical Sciences and Peking Union Medical College, Beijing, China; 3 Department of Pulmonary Medicine, Shanghai Chest Hospital, Shanghai Jiao Tong University, Shanghai, China; 4 Department of Radiology, Henan Cancer Hospital, The Affiliated Cancer Hospital of Zhengzhou University, Zhengzhou, China

## Abstract

**Importance:**

Lung cancer screening has been widely implemented in Europe and the US. However, there is little evidence on participation and diagnostic yields in population-based lung cancer screening in China.

**Objective:**

To assess the participation rate and detection rate of lung cancer in a population-based screening program and the factors associated with participation.

**Design, Setting, and Participants:**

This cross-sectional study used data from the Cancer Screening Program in Urban China from October 2013 to October 2019, with follow-up until March 10, 2020. The program is conducted at centers in 8 cities in Henan Province, China. Eligible participants were aged 40 to 74 and were evaluated for a high risk for lung cancer using an established risk score system.

**Main Outcomes and Measures:**

Overall and group-specific participation rates by common factors, such as age, sex, and educational level, were calculated. Differences in participation rates between those groups were compared. The diagnostic yield of both screening and nonscreening groups was calculated.

**Results:**

The study recruited 282 377 eligible participants and included 55 428 with high risk for lung cancer; the mean (SD) age was 55.3 (8.1) years, and 34 966 participants (63.1%) were men. A total of 22 260 participants underwent LDCT (participation rate, 40.16%; 95% CI, 39.82%-40.50%). The multivariable logistic regression model showed that female sex (odds ratio [OR], 1.64; 95% CI, 1.52-1.78), former smoking (OR, 1.26; 95% CI, 1.13-1.41), lack of physical activity (OR, 1.19; 95% CI, 1.14-1.24), family history of lung cancer (OR, 1.73; 95% CI, 1.66-1.79), and 7 other factors were associated with increased participation of LDCT screening. Overall, at 6-year follow-up, 78 participants in the screening group (0.35%; 95% CI, 0.29%-0.42%) and 125 in the nonscreening group (0.38%; 95% CI, 0.33%-0.44%) had lung cancer detected, which resulted in an odds ratio of 0.93 (95% CI, 0.70-1.23; *P* = .61).

**Conclusions and Relevance:**

The low participations rate in the program studied suggests that an improved strategy is needed. These findings may provide useful information for designing effective population-based lung cancer screening strategies in the future.

## Key Points

**Question:**

What were the participation rate and detection rate of lung cancer and the factors associated with participation in a population-based screening program in China?

**Findings:**

In this cross-sectional study of 282 377 participants including 55 428 with high risk for lung cancer, adherence to low-dose computed tomography screening was 40.16%, and factors associated with the willingness to accept low-dose computed tomography screening included female sex, former smoking, lack of physical activity, and family history of lung cancer.

**Meaning:**

These findings may inform future evaluations of the effectiveness and cost-effectiveness of cancer screening programs in China.

## Teaser

This cross-sectional study assesses the participation and detection rates of a lung cancer screening program in China and the factors associated with participation and detection among individuals at high risk for lung cancer.

## Introduction

Lung cancer is the leading cause of death from cancer worldwide. According to the World Health Organization, the number of deaths due to lung cancer worldwide in 2018 was approximately 1.76 million, accounting for 18.4% of all deaths from cancer.^1^ In China, according to the report of the Third National Mortality Retrospective Sampling Survey, the lung cancer mortality rate has increased by 465% in the past 30 years.^2^ Although some progress has been made in lung cancer treatment in recent years, the prognosis of lung cancer has not improved significantly, and the current 5-year survival rate in China is only 19.7%.^3^ It is well known that if surgical resection can be performed at an early stage (especially stage I), the prognosis of lung cancer will be significantly improved.^4^ Low-dose computed tomography (LDCT) of the chest is currently recognized as an imaging test associated with reduced lung cancer mortality in high-risk populations.^4^ The National Lung Screening Trial (NLST)^4^ demonstrated a 20% reduction in lung cancer mortality associated with LDCT screening of high-risk individuals compared with chest radiography screening in 2011.

When evaluating the effect of screening methods in the population, in addition to focusing on diagnostic-related indicators such as sensitivity, specificity, predictive value, and likelihood ratio, the target population's compliance with the screening method requires attention. However, data on compliance rates for LDCT in population-based screening programs are still sparse.

In October 2012, the National Health Committee of China announced the launch of the Cancer Screening Program in Urban China (CanSPUC), which targets 6 types of cancer that are most prevalent in urban areas, including lung cancer, female breast cancer, esophageal cancer, gastric cancer, colorectal cancer, and liver cancer.^5^ Eligible participants are recruited in the communities of the study regions and are invited to undergo cancer screening free of charge. Participants are first invited to take a cancer risk assessment using an established clinical cancer risk score system, and those who are evaluated to be at high risk for specific types of cancer are recommended to take the appropriate screening intervention per the study protocol. For lung cancer screening, participants at high risk for lung cancer are recommended to undergo subsequent LDCT at tertiary-level hospitals designated by the program.

For the present study, we report the results of lung cancer screening conducted in the first 6 years of this cancer screening program in Henan Province, China, between October 2013 and October 2019. We assessed the participation rate and diagnostic yield of LDCT screening in high-risk populations in China at 6 years of follow-up with the aim to provide references for designing effective lung cancer screening strategies in the future.

## Methods

### Study Design and Participants

We performed a cross-sectional study under the framework of CanSPUC. The study methods are described elsewhere.^5^ In brief, residents aged 40 to 74 years living in the selected communities of the 8 participating cities were approached by trained staff by means of telephone calls and personal encounter. Social media and community advertisement were used to raise the public awareness of this cancer screening program. All the eligible participants were interviewed by trained staff to collect information about their exposure to risk factors and to evaluate their cancer risk using an established risk score system. For the present screening program, to optimize use of the limited health care resources and to enhance the detection rate of lung cancer, only participants who were assessed to be at high risk of lung cancer were recommended to undergo LDCT examination free of charge at a tertiary-level hospital designated by the program. The present study was approved by the Ethics Board of Henan Cancer Hospital, including a waiver for patient consent because all personally identifiable information was removed from the data sets. This study followed the Strengthening the Reporting of Observational Studies in Epidemiology (STROBE) reporting guideline.

For the present analyses, we used data from the lung cancer screening conducted in the first 6 years between October 2013 and October 2019 in Henan Province, which covered a total of 8 cities (Zhengzhou, Zhumadian, Anyang, Luoyang, Nanyang, Jiaozuo, Puyang, and Xinxiang). Overall, 282 377 eligible participants were recruited. After excluding participants with invalid risk assessment results (n = 2) and those not at high risk for lung cancer (n = 226 947), 55 428 remaining participants were included in the present study. A flow diagram showing the recruitment of the study population is shown in eFigure 1 in the Supplement.

### Risk Assessment

Participants were required to undergo risk assessment before LDCT. The rationale of the development of the cancer risk score system followed the Harvard Risk Index,^6^ but the included risk factors, relative risks, and exposure rates of risk factors were adjusted according to the characteristics of the Chinese population. Each risk factor was allocated a score by the expert panel based on the magnitude of its association with lung cancer. The cumulative risk scores were calculated and were then divided by the average risk score in the general population to get the final individual relative risks. Individuals with a relative risk over 1.50 or age 50 years or older and smoking index of 400 or greater (number of cigarettes smoked per day multiplied by years of smoking) were defined as being at high risk for lung cancer.

### LDCT Scanning

All participants underwent LDCT using a 16-section multidetector CT machine (LightSpeed-16; General Electric Company). The protocol parameters were 120 KVp and 30 mAs for LDCT, 512 × 512 matrix, field of view 400 mm ×400 mm or 500 mm ×500 mm, collimation 128 × 0.625 mm or 16 × 1.25 mm, rotation 0.5 seconds, pitch 0.8 or 1.02, 1.25-mm section width with a 1.25-mm reconstruction interval, and duration of scan 3 to 10 seconds. Unenhanced spiral acquisitions were obtained with a breath hold from the thoracic inlet to lung bases with images. Images were reconstructed using a standard algorithm. All images were sent to a General Electric Advantage 4.6 Workstation and underwent multiplanar reconstruction. All studies were reviewed on a PACS workstation (NEUsoft) with the window level of −500 to −700 HU and width of 1400 HU.

### Data Acquisition

Paper-based standardized documentation forms (epidemiological questionnaire, LDCT report) were collected from trained staff and physicians. Validity of forms was checked and entered into the data management system by trained study staff. A consistency check was conducted, and mistakes were corrected by retrieving the original records if inconsistencies were identified. Each participant had a unique identification code that was used to track all the individual’s relevant documentation forms. All data were transmitted to the Central Data Management Team in the National Cancer Center of China, where the databases were constructed and analyses were performed.

### Follow-up Data

All new cases of lung cancer in the study were ascertained through local cancer registry databases on the basis of a histologically confirmed diagnosis from October 1, 2013, to March 10, 2020, in mainland China. Newly diagnosed cases of lung cancer were classified by sites according to *International Statistical Classification of Diseases and Related Health Problems, Tenth Revision* (codes C33 and C34).

### Statistical Analysis

In addition to the descriptive analyses regarding the characteristics of the study population, overall and group-specific participation rates by common factors were calculated; respective 95% CI are reported. Differences in participation rates between groups were compared using the χ^2^ test. Associations of factors with participation rate in LDCT were quantified by odds ratios (ORs) and their 95% CIs, which were derived from multivariable logistic regression models after adjustment for ethnicity, occupation, recruitment, and study sites. Factors studied included age; sex; race; occupation; body mass index; educational background; marriage status; smoking status; alcohol consumption; physical activity; history of tuberculosis, chronic bronchitis, emphysema, and asthma bronchiectasis, and family history of lung cancer. Diagnostic yield of both screening and nonscreening groups, including detection rates of location and histologic type of lung cancer, was calculated. Associations of various characteristics with prevalence of lung cancer were likewise quantified by ORs and their 95% CIs using logistic regression models. All statistical analyses were performed using SAS, version 9.4 (SAS Institute). All tests were 2-sided, and *P* ≤ .05 was considered to be statistically significant.

## Results

### Characteristics of the Study Population

Characteristics of the population at high risk of lung cancer are presented in Table 1. Overall, more men (34 966 [63.1%]) were included in the study. The mean (SD) age was 55.3 (8.1) years, and most participants (41 161 [74.3%]) were aged 45 to 64 years.

**Table 1.** Characteristic of the Study Population and Participation Rates

Characteristic	Participants, No. (%)		Participation rate, %	χ^2^	*P* value
	At high risk for lung cancer	Underwent LDCT^a^			
City					
Zhengzhou	34 044 (61.42)	13 255 (59.55)	38.93	450.37	<.001
Zhumadian	5956 (10.75)	1890 (8.49)	31.73		
Anyang	10 696 (19.30)	4932 (22.16)	46.11		
Luoyang	1124 (2.03)	513 (2.30)	45.64		
Nanyang	2412 (4.35)	1065 (4.78)	44.15		
Jiaozuo	510 (0.92)	241 (1.08)	47.25		
Puyang	305 (0.55)	179 (0.80)	58.69		
Xinxiang	381 (0.69)	185 (0.83)	48.56		
Age, y					
40-44	5511 (9.94)	2148 (9.65)	38.98	46.88	<.001
45-49	9908 (17.88)	3935 (17.68)	39.72		
50-54	11 479 (20.71)	4712 (21.17)	41.05		
55-59	9946 (17.94)	4080 (18.33)	41.02		
60-64	9828 (17.73)	4007 (18.00)	40.77		
65-69	6579 (11.87)	2638 (11.85)	40.10		
70-74	2177 (3.93)	740 (3.32)	33.99		
Sex					
Male	34 966 (63.08)	11 847 (53.22)	33.88	1553.76	<.001
Female	20 462 (36.92)	10 413 (46.78)	50.89		
Race					
Han	54 172 (97.73)	21 774 (97.82)	40.19	1.15	.28
Others	1256 (2.27)	486 (2.18)	38.69		
Occupation					
Farmer	11 132 (20.08)	4446 (19.97)	39.94	99.95	<.001
Enterprise or company employee or worker	25 218 (45.50)	10 039 (45.10)	39.81		
Self-employed or unemployed	9415 (16.99)	3733 (16.77)	39.65		
Public sector employee	7537 (13.60)	3303 (14.84)	43.82		
Other	2126 (3.84)	739 (3.32)	34.76		
BMI					
<18.5	772 (1.39)	295 (1.33)	38.21	4.68	.20
18.5-24.0	22 033 (39.75)	8762 (39.36)	39.77		
24.0-28.0	25 168 (45.41)	10 154 (45.62)	40.34		
≥28.0	7455 (13.45)	3049 (13.7)	40.90		
Educational level					
Primary school or less	8636 (15.58)	3304 (14.84)	38.26	108.91	<.001
Junior or senior high school	37 996 (68.55)	14 992 (67.35)	39.46		
Undergraduate degree or more	8796 (15.87)	3964 (17.81)	45.07		
Marriage					
Unmarried, divorce, or widowed	2125 (3.83)	937 (4.21)	44.09	14.23	<.001
Married	53 303 (96.17)	21 323 (95.79)	40.00		
Smoking status					
Never	17 257 (31.13)	8752 (39.32)	50.72	1256.70	<.001
Current	35 941 (64.84)	12 500 (56.15)	34.78		
Former	2230 (4.02)	1008 (4.53)	45.20		
Alcohol consumption					
Never	24 972 (45.05)	10 436 (46.88)	41.79	50.40	<.001
Current	27 195 (49.06)	10 549 (47.39)	38.79		
Former	3261 (5.88)	1275 (5.73)	39.10		
Physical activity, times/wk					
<3	40 051 (72.26)	16 855 (75.72)	42.08	222.30	<.001
≥3	15 377 (27.74)	5405 (24.28)	35.15		
History of tuberculosis					
No	52 375 (94.49)	20 492 (92.06)	39.13	423.59	<.001
Yes	3053 (5.51)	1768 (7.94)	57.91		
History of chronic bronchitis					
No	28 399 (51.24)	9137 (41.05)	32.17	1545.73	<.001
Yes	27 029 (48.76)	13 123 (58.95)	48.55		
History of emphysema					
No	52 187 (94.15)	20 489 (92.04)	39.26	300.47	<.001
Yes	3241 (5.85)	1771 (7.96)	54.64		
History of asthma bronchiectasis					
No	47 331 (85.39)	18 003 (80.88)	38.04	608.14	<.001
Yes	8097 (14.61)	4257 (19.12)	52.58		
Family history of lung cancer					
No	37 656 (67.94)	12 702 (57.06)	33.73	2019.60	<.001
Yes	17 772 (32.06)	9558 (42.94)	53.78		

Abbreviations: BMI, body mass index (calculated as weight in kilograms divided by height in meters squared); LDCT, low-dose computed tomography.

### Participation Rate for Screening LDCT and Associated Factors

Of the 55 428 participants at high risk for lung cancer, 22 260 underwent LDCT as recommended by the program. The overall participation rate was 40.16% (95% CI, 39.82%-40.50%). The participation rates stratified by potential associated factors are shown in Table 1. Overall, compared with the lowest participation rate in Zhumadian (31.7%), Puyang City had the highest participation rate (58.7%). The participation rates were higher among female than male participants (50.9% vs 33.9%, *P* < .001) and among participants aged 50 to 69 years compared with the other age groups. Univariate analyses showed that participants who were public sector employees; had a high educational level; were unmarried, divorce, or widowed; never smoked; never consumed alcohol; lacked physical activity; had a history of tuberculosis, chronic bronchitis, emphysema, asthma bronchiectasis; and had a family history of lung cancer had higher participation rates.

We also conducted multivariable logistic regression models to explore the potential factors associated with participation rate, and the results are shown in Table 2. We found that age; sex; educational level; smoking status; alcohol consumption status; physical activity; history of tuberculosis, chronic bronchitis, emphysema, and asthma bronchiectasis; and family history of lung cancer were associated with participation rate. For instance, the odds of participants with a history of chronic bronchitis undergoing LDCT screening were 40% higher odds than that of participants with no history of chronic bronchitis (OR, 1.42; 95% CI, 1.36-1.47). Participants with a family history of lung cancer who underwent LDCT screening had a 0.7 higher odds of undergoing screening than did participants with no family history of lung cancer (OR, 1.73; 95% CI, 1.66-1.79). Because participation rates varied among the study sites and years of participant recruitment, these 2 factors were additionally analyzed in the adjusted model, and the ORs did not change greatly (Table 2).

**Table 2.** Factors Associated With Participation Rate in Low-Dose Computed Tomography in the Screening Program

Factors	Model 1^a^		Model 2^b^	
	OR (95% CI)	*P* value	OR (95% CI)	*P* value
Age, y				
40-44	1 [Reference]	NA	1 [Reference]	NA
45-49	1.08 (1.01-1.16)	.03	1.09 (1.02-1.18)	.01
50-54	1.16 (1.08-1.24)	<.001	1.19 (1.11-1.28)	<.001
55-59	1.20 (1.12-1.29)	<.001	1.24 (1.16-1.33)	<.001
60-64	1.21 (1.13-1.30)	<.001	1.26 (1.17-1.35)	<.001
65-69	1.20 (1.11-1.30)	<.001	1.25 (1.15-1.35)	<.001
70-74	0.89 (0.80-1.00)	.04	0.90 (0.80-1.00)	.05
Sex				
Male	1 [Reference]	NA	1 [Reference]	NA
Female	1.64 (1.52-1.78)	<.001	1.69 (1.56-1.83)	<.001
Educational level				
Primary school or less	1 [Reference]	NA	1 [Reference]	NA
Junior or senior high school	1.14 (1.08-1.20)	<.001	1.14 (1.08-1.20)	<.001
Undergraduate degree or more	1.36 (1.27-1.47)	<.001	1.34 (1.24-1.44)	<.001
Smoking status				
Never	1 [Reference]	NA	1 [Reference]	NA
Current	0.96 (0.89-1.05)	.37	0.97 (0.89-1.06)	.49
Former	1.26 (1.13-1.41)	<.001	1.27 (1.14-1.42)	<.001
Alcohol consumption				
Never	1 [Reference]	NA	1 [Reference]	NA
Current	1.21 (1.16-1.27)	<.001	1.23 (1.17-1.29)	<.001
Former	1.24 (1.15-1.35)	<.001	1.24 (1.14-1.35)	<.001
Physical activity, times/wk				
<3	1.19 (1.14-1.24)	<.001	1.20 (1.15-1.25)	<.001
≥3	1 [Reference]	NA	1 [Reference]	NA
History of tuberculosis				
No	1 [Reference]	NA	1 [Reference]	NA
Yes	1.53 (1.42-1.66)	<.001	1.47 (1.36-1.59)	<.001
History of chronic bronchitis				
No	1 [Reference]	NA	1 [Reference]	NA
Yes	1.42 (1.36-1.47)	<.001	1.47 (1.41-1.53)	<.001
History of emphysema				
No	1 [Reference]	NA	1 [Reference]	NA
Yes	1.18 (1.10-1.28)	<.001	1.18 (1.09-1.27)	<.001
History of asthma bronchiectasis				
No	1 [Reference]	NA	1 [Reference]	NA
Yes	1.18 (1.12-1.25)	<.001	1.21 (1.15-1.28)	<.001
Family history of lung cancer				
No	1 [Reference]	NA	1 [Reference]	NA
Yes	1.73 (1.66-1.79)	<.001	1.71 (1.64-1.78)	<.001

Abbreviation: OR, odds ratio; NA, not applicable.

^a^ Odds ratios were adjusted for factors including occupation; age; sex; race/ethnicity; body mass index; educational level; smoking status; alcohol consumption; physical activity; history of tuberculosis, chronic bronchitis, emphysema, or asthma bronchiectasis; and family history of lung cancer in the logistic regression model.

^b^ In addition to the factors included in model 1, ORs were additional for year of recruitment and study sites in the logistic regression model.

### Lung Cancer in Screening and Nonscreening Groups

Table 3 and eFigure 2 in the Supplement show the detection rates of lung cancer according to a follow-up period of March 10, 2020. At 6-year follow-up, the detection rate of lung cancer was 0.35% (78 cases; 95% CI, 0.29%-0.42%) in the screening group and 0.38% (125 cases; 95% CI, 0.33%-0.44%) in the nonscreening group, which resulted in an OR of 0.93 (95% CI, 0.70-1.23; *P* = .61). Similar ORs, which were not significantly different between the 2 groups, were observed for clear location and histologic type. For unclear location and histologic type, we observed a low detection rate of lung cancer in the screening group, with ORs of 0.61 (95% CI, 0.41-0.92) for unclear location and 0.36 (95% CI, 0.18-0.75) for unclear histologic types.

**Table 3.** Lung Cancer Location and Histologic Type Until the Data Cutoff Date of March 10, 2020

Variable	Group, No. (%)		OR (95% CI)	*P* value
	Screening (n = 78)	Nonscreening (n = 125)		
Location				
Upper	24 (0.11)	23 (0.07)	1.55 (0.88-2.76)	.13
Middle	5 (0.02)	5 (0.02)	1.49 (0.43-5.15)	.53
Lower	14 (0.06)	14 (0.04)	1.49 (0.71-3.13)	.29
Others	2 (0.01)	3 (0.01)	0.99 (0.17-5.94)	>.99
Unclear	33 (0.15)	80 (0.24)	0.61 (0.41-0.92)	.02
Histologic type				
Adenocarcinoma	48 (0.22)	51 (0.15)	1.40 (0.95-2.08)	.09
Squamous cell carcinoma	10 (0.04)	24 (0.07)	0.62 (0.30-1.30)	.21
Small-cell carcinoma	6 (0.03)	9 (0.03)	0.99 (0.35-2.79)	>.99
Others	5 (0.02)	4 (0.01)	1.86 (0.50-6.94)	.35
Unclear	9 (0.04)	37 (0.11)	0.36 (0.18-0.75)	.006
Total	78 (0.35)	125 (0.38)	0.93 (0.70-1.23)	.61

Abbreviation: OR, odds ratio.

**Figure 1.**
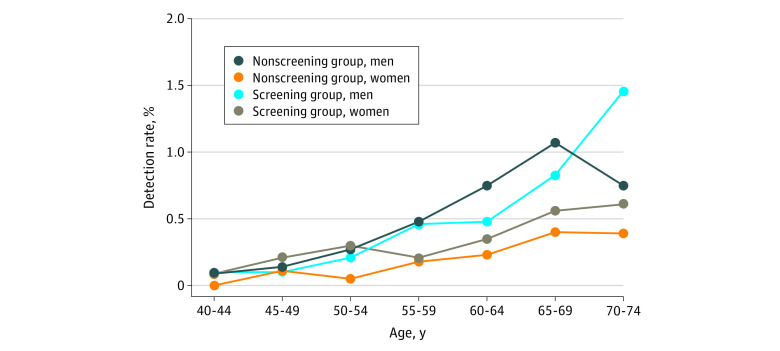
Detection Rates of Lung Cancer in the Screening and Nonscreening Groups Stratified by Age and Sex

The detection rates for lung cancer increased with increasing age and were higher among participants male than among female participants in the screening and nonscreening groups (Figure 1). For instance, the detection rate of lung cancer among men aged 70 to 74 years was 1.46% (95% CI, 0.64%-2.87%) in the screening group and 0.75% (95% CI, 0.35%-1.41%) in nonscreening group. These rates were significantly higher than the rates for women in the same age range (0.61%; 95% CI, 0.11%-1.90%) in screening group and 0.39% (95% CI, 0.07%-1.24%) in nonscreening group.

### Factors Associated With Lung Cancer Detection

**Figure 2.**
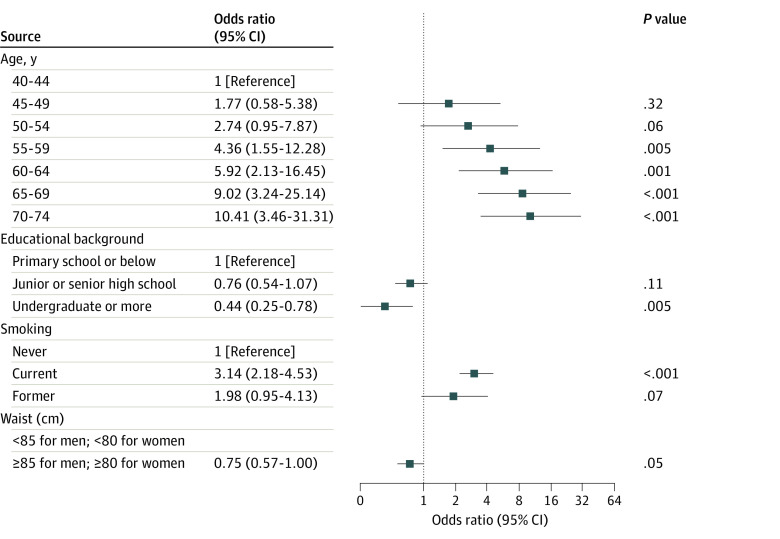
Forest Plot of Factors Associated With Lung Cancer

Older age and current smokers were identified to be positively associated with lung cancer (Figure 2). High educational level and larger waist were identified to be protectively associated with lung cancer. For instance, compared with individuals aged 40 to 44 years, the OR for individuals aged 70 to 74 years having lung cancer was 10.41 (3.46-31.31).

## Discussion

The study reported the results of 55 428 participants who underwent lung cancer screening in a population-based cancer screening program in China. To our knowledge, our study is the first to present the participation rates and diagnostic yield of lung cancer screening using a strategy combining risk score stratification and LDCT based on results from a large-scale cancer screening program in China.

The study found that the overall participation rate (40.16%) in LDCT screening for high-risk populations in urban China still needs to be improved. There were certain regional differences, which may be related to the mobilization organization, publicity and education, and service capabilities of the communities and hospitals at the participating cities. The NLST,^4^ which began in 2002, has a 95% participation rate in high-risk populations and is one of the few randomized clinical trials with high compliance. On the basis of the LDCT screening result of NLST, the US Preventive Services Task Force and the Centers for Medicare & Medicaid Services approved recommendations for lung cancer screening, allowing access to patients at no cost. However, according to the National Health Interview Survey, among 6.8 million eligible patients, only 260 000 received LDCT screening (3.8%) in 2015.^7^ The poor compliance with LDCT screening appears to be a common problem in real-world LDCT screening programs involving large sample populations.

In the CanSPUC study, the overall participation rates in lung cancer screening were higher than rates in colorectal cancer screening by colonoscopy (14.0%)^8^ and upper gastrointestinal cancer screening by gastroscopy (18.4%).^5^ A history of tuberculosis, chronic bronchitis, emphysema, or asthma bronchiectasis and a family history of lung cancer are risk factors for lung cancer, as confirmed by research.^9,10,11,12^ This study found that people with these characteristics had better LDCT screening compliance. From a clinical perspective, pulmonary tuberculosis, chronic bronchitis, emphysema, and asthma bronchiectasis usually require LDCT to confirm the diagnosis, and clinicians recommend that these high-risk populations be regularly reviewed for LDCT. High-risk populations with a family history of lung cancer may have a higher recognition of the importance of lung cancer screening. In addition, the participation rate of LDCT screening among people aged 40 to 44 years and 70 to 74 years, who were male, who had a lower educational level, and who were current smokers was low. The underlying reasons may have been the long time gap between recruitment and actual LDCT screening (median, 0.96 months), long distance to a screening hospital, and poor awareness and knowledge about lung cancer screening. However, factors that were associated with nonparticipation were not evaluated in our study and needed to be further explored. Our results suggest that public awareness campaigns are necessary to improve the participation rate of lung cancer screening in the future.

The overall lung cancer detection rates at 6 years of follow-up in screening and nonscreening groups were close at 0.35% and 0.38%, respectively. The results were similar to the results from German Lung Cancer Screening Intervention, which found detection rates of 0.79% and 1.04% in the LDCT screening group and the control group, respectively, with a mean follow-up time of 8.8 years by linkage to a cancer registry.^13^ However, the detection rates were lower than the overall findings. Therefore, the low detection rate in our study might be explained by patients only being screened once, whereas early lung cancer sometimes require serial scans to be clinically apparent.

Our study showed that several sociodemographic factors, including age, low level of education, and smoking, were positively associated with lung cancer in this high-risk population. The associations of these factors with lung cancer have been extensively explored in the general population, and our findings are in lines with those of previous studies.^14,15^

Of note, our study found that the overall detection rate for lung cancer in the screening group was only slightly lower than in the nonscreening group. Given the relatively low participation rate in LDCT screening, most lung cancer cases were missed during the program, which substantially reduced the effectiveness of screening. To further improve the diagnostic yield of lung cancer screening in China, the following issues should be addressed in next-step research: optimize the risk assessment score based on the current study findings and other well-established risk prediction scores^16,17,18,19^; explore the role of less harmful tests as a supplement to LDCT screening; design novel risk-adapted screening strategies covering both high-risk and low-risk populations using appropriate screening modalities; and perform multifactor interventions targeting multiple levels of care with the purpose of optimizing lung cancer screening acceptance.

### Strengths and Limitations

This study has strengths. To our knowledge, these analyses were the first to show the participation and diagnostic yield of LDCT screening in a large-scale population-based cancer screening program in China. Furthermore, detailed patient information including epidemiological questionnaire and clinical examination data were collected in a standardized manner by trained study staff to ensure the quality of data. Capacity training and central review of LDCT reports by an expert panel were also conducted yearly to enhance the consistency and accuracy of clinical diagnoses.

This study also has limitations. Although the study population was selected from 8 cities, our study may be not representative of the entire general population of Henan Province, and therefore selection bias cannot be ruled out. Second, our study did not collect information about the reasons for nonparticipantion and lost part of the subjective information on nonparticipants. Third, given that follow-up work for patients diagnosed with lung cancer is still under way, clinical disease information was not fully obtained. Therefore, tumor stage information was not reported in our study.

## Conclusions

In cross-sectional study, we found a low participation rate in cancer screening. We further identified several factors associated with the participation rate in LDCT screening and risk factors of lung cancer. Our findings may provide important references for designing effective population-based lung cancer screening strategies in the future.

## Notes

Supplement. **eFigure 1.** Flow Diagram of Participant Recruitment in CanSPUC, 2013-2019 **eFigure 2.** Anatomical Location of Lung Cancers Between the Screening Group and Non-Screening Group Until the Data-Cutoff Date of March 10, 2020

## References

[1] Ferlay J, Colombet M, Soerjomataram I, Estimating the global cancer incidence and mortality in 2018: GLOBOCAN sources and methods. Internat J Cancer. 2018. 30350310 10.1002/ijc.31937

[2] Chen Z. Report of the Third National Mortality Retrospective Sampling Survey. Peking Union Medical College Press; 2008.

[3] Zeng H, Chen W, Zheng R, Changing cancer survival in China during 2003-15: a pooled analysis of 17 population-based cancer registries. Lancet Glob Health. 2018;6(5):e555-e567. doi:10.1016/S2214-109X(18)30127-X 29653628

[4] Aberle DR, Adams AM, Berg CD, ; National Lung Screening Trial Research Team Reduced lung-cancer mortality with low-dose computed tomographic screening. N Engl J Med. 2011;365(5):395-409. doi:10.1056/NEJMoa1102873 21714641 PMC4356534

[5] Guo L, Zhang S, Liu S, Determinants of participation and detection rate of upper gastrointestinal cancer from population-based screening program in China. Cancer Med. 2019;8(16):7098-7107. doi:10.1002/cam4.2578 31560836 PMC6853828

[6] Colditz GA, Atwood KA, Emmons K, Harvard report on cancer prevention volume 4: Harvard Cancer Risk Index. Risk Index Working Group, Harvard Center for Cancer Prevention. Cancer Causes Control. 2000;11(6):477-488. doi:10.1023/A:1008984432272 10880030

[7] Jemal A, Fedewa SA Lung cancer screening with low-dose computed tomography in the United States-2010 to 2015. JAMA Oncol. 2017;3(9):1278-1281. doi:10.1001/jamaoncol.2016.6416 28152136 PMC5824282

[8] Chen H, Li N, Ren J, Participation and yield of a population-based colorectal cancer screening programme in China. Gut. 2018. 30377193 10.1136/gutjnl-2018-317124

[9] Cheng MP, Abou Chakra CN, Yansouni CP, Risk of active tuberculosis in patients with cancer: a systematic review and meta-analysis. Clin Infect Dis. 2017;64(5):635-644. 27986665 10.1093/cid/ciw838

[10] Lissowska J, Foretova L, Dabek J, Family history and lung cancer risk: international multicentre case-control study in Eastern and Central Europe and meta-analyses. Cancer Causes Control. 2010;21(7):1091-1104. doi:10.1007/s10552-010-9537-2 20306329

[11] Qu YL, Liu J, Zhang LX, Asthma and the risk of lung cancer: a meta-analysis. Oncotarget. 2017;8(7):11614-11620. doi:10.18632/oncotarget.14595 28086224 PMC5355290

[12] Zhang X, Jiang N, Wang L, Liu H, He R Chronic obstructive pulmonary disease and risk of lung cancer: a meta-analysis of prospective cohort studies. Oncotarget. 2017;8(44):78044-78056. doi:10.18632/oncotarget.20351 29100446 PMC5652835

[13] Becker N, Motsch E, Trotter A, Lung cancer mortality reduction by LDCT screening—results from the randomized German LUSI Trial. Int J Cancer. 2020;146(6):1503-1513. doi:10.1002/ijc.32486 31162856

[14] O’Keeffe LM, Taylor G, Huxley RR, Mitchell P, Woodward M, Peters SAE Smoking as a risk factor for lung cancer in women and men: a systematic review and meta-analysis. BMJ Open. 2018;8(10):e021611. doi:10.1136/bmjopen-2018-021611 30287668 PMC6194454

[15] Neal RD, Sun F, Emery JD, Callister ME Lung cancer. BMJ. 2019;365:l1725. doi:10.1136/bmj.l1725 31160279

[16] Etemadi A, Abnet CC, Golozar A, Malekzadeh R, Dawsey SM Modeling the risk of esophageal squamous cell carcinoma and squamous dysplasia in a high risk area in Iran. Arch Iran Med. 2012;15(1):18-21. 22208438 PMC3294378

[17] Hippisley-Cox J, Coupland C Development and validation of risk prediction algorithms to estimate future risk of common cancers in men and women: prospective cohort study. BMJ Open. 2015;5(3):e007825. doi:10.1136/bmjopen-2015-007825 25783428 PMC4368998

[18] Chang J, Huang Y, Wei L, Risk prediction of esophageal squamous-cell carcinoma with common genetic variants and lifestyle factors in Chinese population. Carcinogenesis. 2013;34(8):1782-1786. doi:10.1093/carcin/bgt106 23536576

[19] Iida M, Ikeda F, Hata J, Development and validation of a risk assessment tool for gastric cancer in a general Japanese population. Gastric Cancer. 2018;21(3):383-390. doi:10.1007/s10120-017-0768-8 29043529

