# Supplementary material for: Evaluation of a Low-Dose Computed Tomography Lung Cancer Screening Program in Henan, China
Source: JAMA Netw Open. 2020 Nov 3;3(11):e2019039. doi: 10.1001/jamanetworkopen.2020.19039 (PMC7610188; doi:10.1001/jamanetworkopen.2020.19039)
Supplement: Supplement. — eFigure 1. Flow Diagram of Participant Recruitment in CanSPUC, 2013-2019 eFigure 2. Anatomical Location of Lung Cancers Between the Screening Group and Non-Screening Group Until the Data-Cutoff Date of March 10, 2020 [file jamanetwopen-e2019039-s001.pdf]

Supplemental Online Content

Guo LW, Chen Q, Shen YC, et al. Evaluation of a low-dose computed tomography lung cancer screening program in Henan, China. *JAMA Netw Open*. 2020;3(11):e2019039. doi:10.1001/jamanetworkopen.2020.19039

eFigure 1. Flow Diagram Of Participant Recruitment in CanSPUC, 2013-2019

eFigure 2. Anatomical Location of Lung Cancers Between the Screening Group and Non-Screening Group Until the Data-Cutoff Date of March 10, 2020

This supplemental material has been provided by the authors to give readers additional information about their work.

© 2020 Guo LW et al. *JAMA Network Open.*

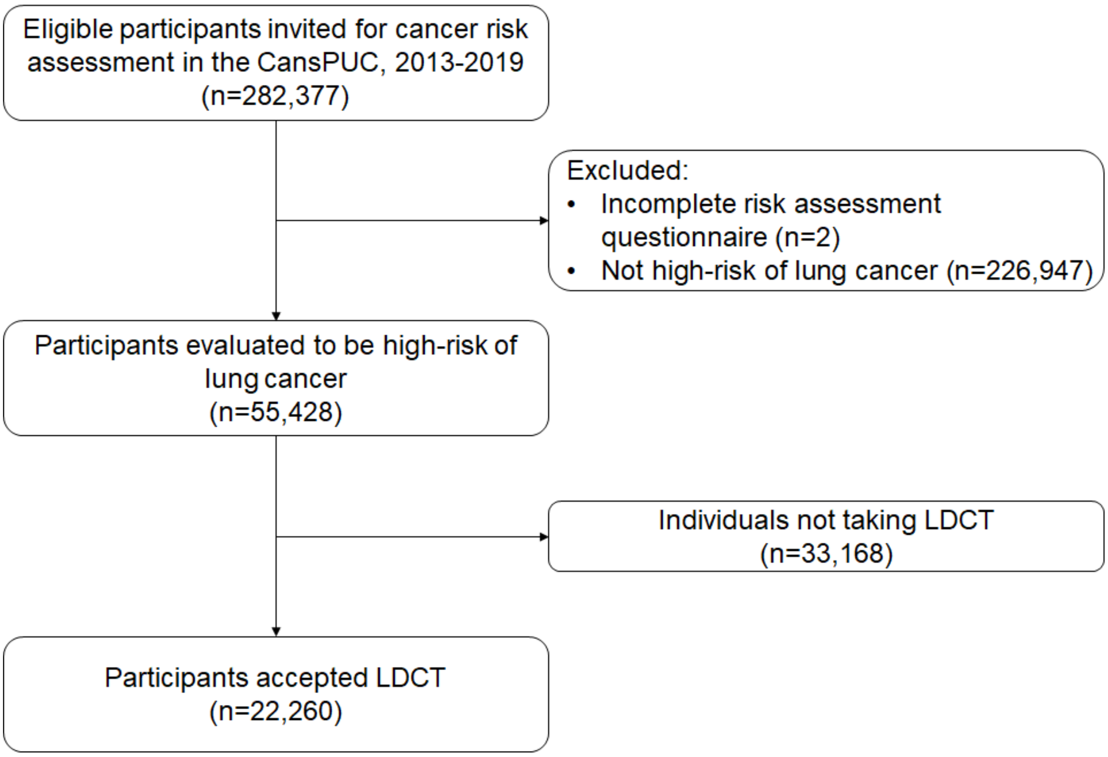

eFigure 1. Flow diagram of participant recruitment in CanSPUC, 2013-2019.

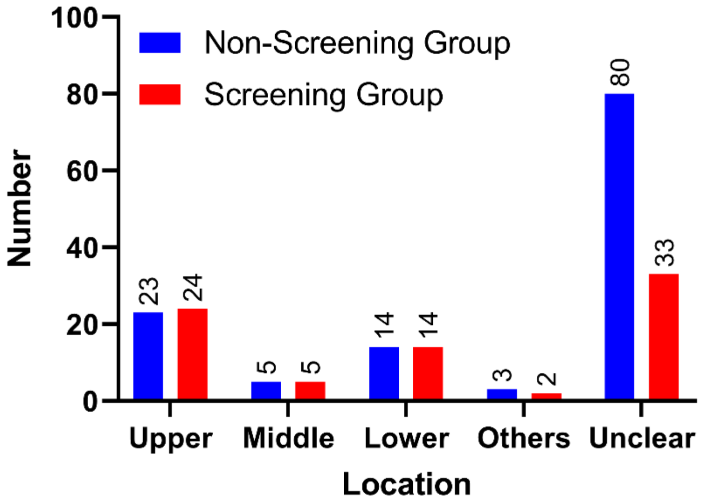

eFigure 2. Anatomical location of lung cancers between the screening group and non-screening group until the data-cutoff date of March 10, 2020.

© 2020 Guo LW et al. *JAMA Network Open.*
